# *IRF7* Gene Variations Confer Susceptibility to Autoimmune Thyroid Diseases and Graves' Ophthalmopathy

**DOI:** 10.1155/2019/7429187

**Published:** 2019-01-15

**Authors:** Qiuming Yao, Xiaofei An, Jing Zhang, Kaida Mu, Ling Li, Ronghua Song, Peilong Sun, Jin-an Zhang

**Affiliations:** ^1^Department of Endocrinology, Jinshan Hospital of Fudan University, No. 1508 Longhang Road, Jinshan District, Shanghai 201508, China; ^2^Department of Endocrinology, The Affiliated Hospital of Nanjing University of Chinese Medicine, Nanjing 210023, China; ^3^Department of Endocrinology, Shanghai University of Medicine & Health Sciences Affiliated Zhoupu Hospital, No. 1500 Zhouyuan Road, Pudong District, Shanghai 201318, China; ^4^Department of General Surgery, Jinshan Hospital of Fudan University, No. 1508 Longhang Road, Jinshan District, Shanghai 201508, China

## Abstract

The objective of this study was to investigate whether *IRF7* polymorphisms are associated with autoimmune thyroid diseases (AITDs). We selected three single nucleotide polymorphisms (SNPs) of *IRF7*, namely, rs1061501, rs1131665, and rs1061502 for genotyping using PCR-based ligase detection reaction (LDR) method in a total of 1659 participants (592 with Graves' disease, 297 with Hashimoto's thyroiditis, and 770 healthy controls). Gene-disease and genotype-clinical phenotype associations were evaluated for the three SNPs. Our results showed that the AG genotype and the minor allele G frequency of rs1131665 and rs1061502 in AITD patients were both higher than those of the controls (rs1131665: AG genotype: *P* = 0.017, OR = 1.968; allele G: *P* = 0.018, OR = 1.946; rs1061502: AG genotype: *P* = 0.029, OR = 1.866; allele G: *P* = 0.031, OR = 1.847). Subgroup analysis also showed that the AG genotype and the minor allele G frequency of rs1131665 and rs1061502 in Graves' disease patients were both higher than those of the controls (rs1131665: AG genotype: *P* = 0.015, OR = 2.074; allele G: *P* = 0.016, OR = 2.048; rs1061502: AG genotype: *P* = 0.034, OR = 1.919; allele G: *P* = 0.035, OR = 1.898). Furthermore, the allele G frequency of rs1061501 was associated with Graves' ophthalmopathy (*P* = 0.035, OR = 1.396). No significant difference in *IRF7* polymorphisms was found between Hashimoto's thyroiditis patients and controls. Our study has revealed for the first time that *IRF7* is a susceptibility gene for AITD, especially for Graves' disease and Graves' ophthalmopathy.

## 1. Introduction

Autoimmune thyroid diseases (AITDs) are a type of T cell-mediated organ-specific autoimmune diseases, the prevalence of which is more than 5% and result from loss of immune tolerance or autoimmune attack to the thyroid itself; they mainly include Graves' disease (GD) and Hashimoto's thyroiditis (HT) [1–3]. The main cause of clinical hyperthyroidism and hypothyroidism is GD and HT, respectively [1, 4]. Hyperthyroidism in GD is attributed to thyroid-stimulating autoantibodies to the TSH receptor, whereas hypothyroidism in HT is related to autoantibodies against thyroid peroxidase and thyroglobulin [5]. The pathogenesis of AITD is thought to be the interaction between susceptibility genes and environmental factors. Recent studies have identified a number of susceptibility loci for AITD, including *CD40* [6], interleukin 10 [7], miR-499a and miR-125a [8], cytotoxic T lymphocyte-associated protein 4 (*CTLA4*), and human leukocyte antigen (*HLA*) [9].

Interferon (IFN) regulatory factors (IRFs), namely, transcriptional regulators of type I IFNs and IFN-inducible genes, play a pivotal role in innate and adaptive immune responses [10]. IRF7, a member of the IRF family, is required for type I IFN production and can regulate type I IFN-dependent immune responses [11]. Recent studies have indicated that polymorphisms in *IRF7* gene are associated with immunity-mediated diseases, systemic lupus erythematosus (SLE) [12, 13] and systemic sclerosis [14]. These studies have contributed to understanding the relationship between the *IRF7* gene mutations and the risk of autoimmune disease, but it is unknown whether *IRF7* gene variations increase or decrease the susceptibility to AITDs.

The purpose of this case-control study was to explore the association of *IRF7* gene polymorphisms with AITDs in a Chinese Han population. Furthermore, we analyzed the correlation between genotypes of *IRF7* gene and AITD clinical phenotypes.

## 2. Materials and Methods

### 2.1. Subjects

A total of 1659 subjects consisting of 889 AITD patients (220 males and 669 females; mean age of 36.29 ± 14.31 years) and 770 controls (216 males and 544 females; mean age of 39.04 ± 8.79 years) were enrolled in this study. The AITD patients were clinically subdivided into 592 GD (179 males and 413 females; mean age of 36.95 ± 14.70 years) and 297 HT (41 males and 256 females; mean age of 34.96 ± 13.42 years). All the AITD patients were recruited from the Outpatient Department of Endocrinology of Jinshan Hospital of Fudan University. GD was diagnosed based on hyperthyroidism and the positive circulating thyroid stimulating hormone receptor antibody (TRAb). HT was defined by high level of positive antibody against thyroid peroxidase (TPOAb) or thyroglobulin (TGAb), with or without hypothyroidism and the presence of an enlarged thyroid.

The controls were healthy individuals recruited from the Check-Up Center of the same hospital. Those controls with any autoimmune disease or family history of thyroid diseases were excluded. All subjects were of Chinese Han ethnicity. The written informed consent was obtained from all the participants. This study was approved by the ethics committee of the hospital.

The clinical phenotypes of AITD patients included the presence or absence of family history within three generations, with or without ophthalmopathy, the onset age (≤18 years vs. ≥19 years), euthyroid status, or hypothyroidism in HT patients.

### 2.2. Genomic DNA Extraction

We obtained 2 mL of peripheral venous blood from all the subjects by venipuncture and then extracted genomic DNA using RelaxGene Blood DNA System (Tiangen Biotech, Beijing, China) according to the manufacturer's guidelines. The concentration and purity of DNA from each sample were detected using NanoDrop 2000 Spectrophotometer (Thermo Scientific Company, Waltham, USA).

### 2.3. SNP Selection and Genotyping

We selected three loci of rs1061501, rs1131665, and rs1061502 in the *IRF7* region because of their strong association with other autoimmune diseases like SLE and systemic sclerosis [13, 14]. Furthermore, marker-tagging SNPs had to meet the criteria of minor allele frequency (MAF) > 0.01, Hardy–Weinberg equilibrium (HWE) with *P* > 0.01, and logarithm of odds (LOD) > 3.0.

The genotyping of three SNPs of *IRF7* was performed using ligase detection reactions (LDR) platform [15–17].

### 2.4. Statistical Analysis

All statistical analyses were performed using SPSS software (version 17.0).

The clinical data of the subjects are presented as M ± SD. The differences in genotype and allele frequency distributions were analyzed by *χ*^2^ test or Fisher's exact test. HWE test, linkage disequilibrium (LD), and haplotype frequency were performed using HaploView 4.2. Odds ratio (OR) and 95% confidence interval (CI) were calculated for the association of genotype or allele frequencies with AITDs.

## 3. Results

### 3.1. Clinical Data Analysis

Clinical data of all the subjects were summarized in Table 1. Our study investigated 889 AITD patients, consisting of 592 GD patients (30.24% males and 69.76% females; mean age of 36.95 ± 14.70 years) and 297 HT ones (13.80% males and 86.20% females; mean age of 34.96 ± 13.42 years). Among the AITDs patients, there were 181 (20.36%) cases with a family history, 111 (12.49%) cases with ophthalmopathy, and 161 (13.72%) cases who were teenager patients with an onset age of ≤18 years old. Among the GD patients, 120 individuals had a family history, 106 had ophthalmopathy, and 83 with an onset age of ≤18 years old (20.27%, 17.91%, and 14.02%, respectively). While in the HT patients, 61 individuals had a family history, 5 had ophthalmopathy and 39 with an onset age of ≤18 years old (20.54%, 1.68%, and 13.13%, respectively).

### 3.2. Allele and Genotypic Results

The genotype distributions of these three SNPs (rs1061501/rs1131665/rs1061502) were in Hardy–Weinberg equilibrium (*P* > 0.05) in both AITD patients and controls (data not shown). Differences in genotypic and allelic frequencies of these three SNPs between AITD patients and controls were compared (Table 2). For rs1131665, the AG genotype frequency and the minor allele G frequency in AITD patients were both significantly higher than those of the controls (AG genotype: 4.5% vs. 2.3%, *P* = 0.017, OR = 1.968, 95% CI = 1.119-3.463; allele G: 2.2% vs. 1.2%, *P* = 0.018, OR = 1.946, 95% CI = 1.111-3.409). Similar trend was also found for rs1061502. However, no significant differences in both allele and genotype frequencies of rs1061501 were found between AITD patients and the controls.

As shown in Table 3, subgroup analysis showed that the AG genotype frequency of rs1131665 and rs1061502 in GD patients was significantly higher than that of the controls (*P* = 0.015, OR = 2.074, 95% CI = 1.136-3.787; *P* = 0.034, OR = 1.919, 95% CI = 1.042-3.535, respectively). The minor allele G frequency of rs1131665 and rs1061502 in GD patients was also significantly higher than that of the controls (*P* = 0.016, OR = 2.048, 95% CI = 1.127-3.721; *P* = 0.035, OR = 1.898, 95% CI = 1.036-3.479, respectively). No significant difference in the three SNPs, however, was found between HT patients and the controls.

### 3.3. Genotype and Clinical Phenotype Correlations

We also analyzed the association of genotypes with clinical phenotypes, including age of onset (≤18 years old or ≥19 years old) in AITD patients, with or without ophthalmopathy in GD patients, hypothyroidism, or euthyroidism in HT patients.

Table 4 displayed that the allele G frequency of rs1061501 in GD patients with ophthalmopathy was significantly higher than that of GD patients without ophthalmopathy (*P* = 0.035, OR = 1.396, 95% CI = 1.023-1.906). As shown in Tables 5 and 6, we did not find any difference in specific genotype frequency or allele frequency in AITD patients with different age of disease onset, also in HT patients accompanied with hypothyroidism or not (all *P* > 0.05).

## 4. Discussion

To our knowledge, the present study was the first to investigate the association of *IRF7* polymorphisms with the susceptibility to AITDs. Our results showed that AG genotype and the minor G allele of rs1131665 and rs1061502 significantly increased the risk of AITDs and GD. More importantly, the minor G allele of rs1061501 was associated with ophthalmopathy in GD patients.

In concordance with our study, Fu et al. have shown that the polymorphisms of rs1131665 in *IRF7* conferred susceptibility to the development of SLE in multiple ethnic groups, including Asians, Chinese, and European Americans. Moreover, their meta-analysis provided direct genetic evidence that *IRF7* is a risk gene for human SLE [18]. Another study involving 2316 SSc patients and 2347 healthy controls demonstrated a significant association of rs1131665 polymorphisms with the presence of anticentromere autoantibodies (ACA) in SSc patients [14]. The TT genotype and T allele of rs1061501 in *IRF7* were also found to be associated with an increased risk of developing SLE among Taiwanese patients, but no significant difference in rs1061502 SNP was found between SLE patients and controls [13]. One possible explanation is that their small sample size (92 SLE patients and 92 healthy controls) caused lower statistical power.

*IRF7* is localized on human chromosome 11p15.5 and interacts with the MyD88 adaptor protein downstream of Toll-like receptor (TLR) signaling [19]. *IRF7* can induce a large amount of type I IFN after being activated by TLR7 and TLR9 in plasmacytoid dendritic cells [20]. Interferon- (INF-) *α*, a type 1 INF, is widely used to treat chronic hepatitis C for its antiviral effects [21]. Furthermore, INF-*α* is also strongly linked with the occurrence of AITDs because it can evoke antibody-mediated immune responses [22, 23]. One study revealed that overt AITD occurred in about 5–10% of INF-*α*-treated patients [24]. A recent study has also found that 2% of patients developed hyperthyroidism in the HCV-positive patients during a 12-week course of INF-*α* antiviral therapy [25]. Therefore, studies have indicated that INF-*α* is one of the important immune factors that induce AITDs [26, 27].

Our results demonstrated that the AG genotype and minor allele G frequency of rs1131665 and rs1061502 in AITD patients and GD patients were both significantly higher than those of the controls, suggesting that minor allele G may be a risk factor for AITD and GD. However, neither the genotype nor allele of rs1131665, rs1061501, and rs1061502 was found to be associated with HT, indicating that the three SNPs were not involved in the pathogenesis of HT. These different results of the *IRF7* SNPs in GD and HT may be attributed to their different etiology. Intriguingly, the further genotyping-clinical phenotype correlation analysis showed that the allele G frequency of rs1061501 in GD patients with ophthalmopathy was significantly higher than that of GD patients without ophthalmopathy, conferring a significant degree of risk for ophthalmopathy in GD patients. Graves' ophthalmopathy, a common extrathyroidal manifestation of GD, affects 25-50% of GD patients to various extent [28]. To date, its etiology remains unclear. To our knowledge, some genes have been related to Graves' ophthalmopathy, such as thyroid-stimulating hormone receptor (TSHR), *CTLA-4*, *HLA-DRB-1*, and *TNF-α* [29–31]. Our results suggested the association of *IRF7* polymorphisms with Graves' ophthalmopathy, thus adding *IRF7* to the list of predisposing genes of Graves' ophthalmopathy. But according to our existing data, we cannot evaluate the association of IRF7 polymorphisms with the clinical activity, severity, and response to treatment of Graves' ophthalmopathy, which needs to be further studied in the future.

Above all, our study was the first to investigate the association of *IRF7* gene polymorphisms with the susceptibility to AITD. Our study suggests that *IRF7* is a crucial risk factor for AITD and GD. Further researches aiming at identifying the function and mechanism of IRF7 in AITDs are needed to be conducted. Our results are needed to perform in other population. In addition, more attention should be paid to other loci of *IRF7* and the potential roles of *IRF7* in other autoimmune diseases.

## Figures and Tables

**Table 1:** Clinical data of all subjects.

	AITD	GD	HT	Control
*N*	889	592	297	770
*Gender*	—	—	—	—
Male	220 (24.75%)	179 (30.24%)	41 (13.80%)	266 (34.55%)
Female	669 (75.25%)	413 (69.76%)	256 (86.20%)	504 (65.45%)
Age (mean ± s.d.)	36.29 ± 14.31	36.95 ± 14.70	34.96 ± 13.42	39.04 ± 8.79
*Family history*	—	—	—	—
(+)	181 (20.36%)	120 (20.27%)	61 (20.54%)	—
(-)	708 (79.64%)	472 (79.73%)	236 (79.46%)	
*Ophthalmopathy*				
(+)	111 (12.49%)	106 (17.91%)	5 (1.68%)	—
(-)	778 (87.51%)	486 (82.09%)	292 (98.32%)	—
*Onset of age*				—
≤18 years	122 (13.72%)	83 (14.02%)	39 (13.13%)	—
≥19 years	767 (86.28%)	509 (85.98%)	258 (86.87%)	—

AITD, autoimmune thyroid disease; GD, Graves' disease; HT, Hashimoto's thyroiditis.

**Table 2:** Allele and genotype frequencies of *IRF7* SNPs in controls and AITD patients.

SNP	Genotype/allele	Control (%)	AITD (%)	*P*	OR	95% CI
rs1061501	AA	344 (44.7)	423 (47.6)			
	AG	357 (46.4)	376 (42.3)	0.237	—	—
	GG	69 (9.0)	90 (10.1)			
	A	1045 (67.9)	1222 (68.7)	0.590	0.961	0.830-1.112
	G	495 (32.1)	556 (31.3)			

rs1131665	AA	752 (97.7)	849 (95.5)			
	AG	18 (2.3)	40 (4.5)	0.017	1.968	1.119-3.463
	GG	0 (0)	0 (0)			
	A	1522 (98.8)	1738 (97.8)	0.018	1.946	1.111-3.409
	G	18 (1.2)	40 (2.2)			

rs1061502	AA	752 (97.7)	851 (95.7)			
	AG	18 (2.3)	38 (4.3)	0.029	1.866	1.056-3.296
	GG	0 (0)	0 (0)			
	A	1522 (98.8)	1740 (97.9)	0.031	1.847	1.050-3.249
	G	18 (1.2)	38 (2.1)			

AITD, autoimmune thyroid disease; OR, odds ratio; 95% CI, 95% confidence intervals.

**Table 3:** Allele and genotype frequencies of *IRF7* SNPs in GD, HT patients, and controls.

SNP	Control (%)	GD (%)	*P*	OR (95% CI)	HT (%)	*P*	OR (95% CI)
*rs1061501*							
AA	344 (44.7)	281 (47.5)	0.565	—	142 (47.8)	0.074	—
AG	357 (46.4)	258 (43.6)	—	—	118 (39.7)	—	—
GG	69 (9.0)	53 (9.0)	—	—	37 (12.5)	—	—
A	1045 (67.9)	820 (69.3)	0.436	0.937 (0.796-1.103)	402 (67.7)	0.936	1.008 (0.823-1.235)
G	495 (32.1)	364 (30.7)			192 (32.3)		
*rs1131665*							
AA	752 (97.7)	564 (95.3)	0.015	2.074 (1.136-3.787)	285 (96.0)	0.132	1.759 (0.837-3.698)
AG	18 (2.3)	28 (4.7)			12 (4.0)		—
GG	0 (0)	0 (0)			0 (0)		
A	1522 (98.8)	1156 (97.6)	0.016	2.048 (1.127-3.721)	582 (98.0)	0.134	1.743 (0.835-3.642)
G	18 (1.2)	28 (2.4)			12 (2.0)		
*rs1061502*							
AA	752 (97.7)	566 (95.6)	0.034	1.919 (1.042-3.535)	285 (96.0)	0.132	1.759 (0.837-3.698)
AG	18 (2.3)	26 (4.4)			12 (4.0)		—
GG	0 (0)	0 (0)			0 (0)		
A	1522 (98.8)	1158 (97.8)	0.035	1.898 (1.036-3.479)	582 (98.0)	0.134	1.743 (0.835-3.642)
G	18 (1.2)	26 (2.2)			12 (2.0)		

GD, Graves' disease; HT, Hashimoto's thyroiditis; OR, odds ratio; 95% CI, 95% confidence intervals.

**Table 4:** *IRF7* genotype and allele distribution in ophthalmopathy or nonophthalmopathy GD patients.

SNP	GD		*P*	OR (95% CI)
	Ophthalmopathy	Nonophthalmopathy		
rs1061501	—	—	—	—
AA	41 (38.7%)	240 (49.4%)	—	—
AG	52 (49.1)	206 (42.4%)	0.102	—
GG	13 (12.3)	40 (8.2%)	—	—
A	134 (63.2%)	686 (70.6%)	0.035	1.396 (1.023-1.906)
G	78 (32.8%)	286 (29.4%)	—	—
rs1131665	—	—	—	—
AA	104 (99.1%)	460 (97.3%)	—	—
AG	2 (0.9%)	26 (2.7%)	0.128	2.939 (0.687-12.579)
GG	0 (0%)	0 (0%)	—	—
A	210 (98.2%)	946 (97.6%)	0.133	2.886 (0.680-12.253)
G	2 (1.8%)	26 (2.4%)	—	—
rs1061502	—	—		
AA	104 (99.1%)	462 (95.1%)	—	—
AG	2 (0.9%)	24 (4.9%)	0.259	2.701 (0.629-11.610)
GG	0 (0%)	0 (0%)	—	—
A	210 (98.2%)	948 (97.5%)	0.265	2.658 (0.623-11.335)
G	2 (1.8%)	24 (2.5%)	—	—

GD, Graves' disease; OR, odds ratio; 95% CI, 95% confidence intervals.

**Table 5:** Allele and genotype distribution of *IRF7* in AITDs patients with or without early onset age.

SNP	Onset age of AITDs patients		*P*	OR (95% CI)
	≤18	≥19		
rs1061501	—	—	—	—
AA	65 (53.3%)	358 (46.7%)	—	—
AG	48 (39.3)	328 (42.8%)	0.315	—
GG	9 (7.4)	81 (10.6%)	—	—
A	178 (73%)	1044 (68.1%)	0.126	1.266 (0.936-1.712)
G	66 (27%)	490 (31.9%)	—	—
rs1131665	—	—	—	—
AA	115 (94.3%)	734 (95.7%)	—	—
AG	7 (5.7%)	33 (4.3%)	0.477	0.739 (0.319-1.709)
GG	0 (0%)	0 (0%)	—	—
A	237 (97.1%)	1501 (97.8%)	0.483	0.744 (0.326-1.702)
G	7 (2.9%)	33 (2.2%)	—	—
rs1061502	—	—		
AA	115 (94.3%)	736 (96%)	—	—
AG	7 (5.7%)	31 (4%)	0.390	0.692 (0.298-1.608)
GG	0 (0%)	0 (0%)	—	—
A	237 (97.1%)	1503 (98%)	0.395	0.698 (0.304-1.604)
G	7 (2.9%)	31 (2%)	—	—

AITD, autoimmune thyroid disease; OR, odds ratio; 95% CI, 95% confidence intervals.

**Table 6:** *IRF7* genotype and allele distribution in clinical subphenotype of HT patients.

SNP	HT		*P*	OR (95% CI)
	Hypothyroidism	Nonhypothyroidism		
rs1061501	—	—	—	—
AA	94 (48.5%)	48 (46.6%)	—	—
AG	73 (37.6)	45 (43.7%)	0.443	—
GG	27 (13.9)	10 (9.7%)	—	—
A	261 (67.3%)	141 (68.4%)	0.770	0.947 (0.659-1.361)
G	127 (32.7%)	65 (31.6%)	—	—
rs1131665	—	—	—	—
AA	187 (96.4%)	98 (95.1%)	—	—
AG	7 (3.6%)	5 (4.9%)	0.834	1.363 (0.422-4.406)
GG	0 (0%)	0 (0%)	—	—
A	381 (98.2%)	201 (97.6%)	0.836	1.354 (0.424-4.320)
G	7 (1.8%)	5 (2.4%)	—	—
rs1061502	—	—		
AA	187 (96.4%)	98 (95.1%)	—	—
AG	7 (3.6%)	5 (4.9%)	0.834	1.363 (0.422-4.406)
GG	0 (0%)	0 (0%)	—	—
A	381 (98.2%)	201 (97.6%)	0.836	1.354 (0.424-4.320)
G	7 (1.8%)	5 (2.4%)	—	—

HT, Hashimoto's thyroiditis; OR, odds ratio; 95% CI, 95% confidence intervals.

## Data Availability

The data analyzed during this study have been provided in the manuscript and any further information can be made available on request to the corresponding author.

## References

[1] Antonelli A., Ferrari S. M., Corrado A., di Domenicantonio A., Fallahi P. (2015). Autoimmune thyroid disorders. *Autoimmunity Reviews*.

[2] Burch H. B., Cooper D. S. (2015). Management of Graves disease: a review. *JAMA*.

[3] Wiersinga W. M. (2014). Thyroid autoimmunity. *Endocrine Development*.

[4] De Leo S., Lee S. Y., Braverman L. E. (2016). Hyperthyroidism. *The Lancet*.

[5] Thewjitcharoen Y., Krittiyawong S., Porramatikul S. (2017). A study of serum IgG4 levels in the clinical metamorphosis of autoimmune thyroid disease. *Journal of Clinical & Translational Endocrinology*.

[6] Wang D., Chen J., Zhang H., Zhang F., Yang L., Mou Y. (2017). Role of different CD40 polymorphisms in Graves’ disease and Hashimoto’s thyroiditis. *Immunological Investigations*.

[7] Jung J. H., Song G. G., Kim J. H., Choi S. J. (2016). Association of interleukin 10 gene polymorphisms with autoimmune thyroid disease: meta-analysis. *Scandinavian Journal of Immunology*.

[8] Cai T., Li J., An X. (2017). Polymorphisms in MIR499A and MIR125A gene are associated with autoimmune thyroid diseases. *Molecular and Cellular Endocrinology*.

[9] Ramgopal S., Rathika C., Padma M. R. (2018). Interaction of HLA-DRB1^∗^ alleles and CTLA4 (+49 AG) gene polymorphism in autoimmune thyroid disease. *Gene*.

[10] Tamura T., Yanai H., Savitsky D., Taniguchi T. (2008). The IRF family transcription factors in immunity and oncogenesis. *Annual Review of Immunology*.

[11] Honda K., Yanai H., Negishi H. (2005). IRF-7 is the master regulator of type-I interferon-dependent immune responses. *Nature*.

[12] Li P., Cao C., Luan H. (2011). Association of genetic variations in the STAT4 and IRF7/KIAA1542 regions with systemic lupus erythematosus in a northern Han Chinese population. *Human Immunology*.

[13] Lin L. H., Ling P., Liu M. F. (2011). The potential role of interferon-regulatory factor 7 among Taiwanese patients with systemic lupus erythematosus. *The Journal of Rheumatology*.

[14] Carmona F. D., Gutala R., Simeón C. P. (2012). Novel identification of the *IRF7* region as an anticentromere autoantibody propensity locus in systemic sclerosis. *Annals of the Rheumatic Diseases*.

[15] Li L., Ding X., Wang X. (2018). Polymorphisms of IKZF3 gene and autoimmune thyroid diseases: associated with Graves’ disease but not with Hashimoto’s thyroiditis. *Cellular Physiology and Biochemistry*.

[16] Song R. H., Qin Q., Yan N. (2015). Variants in IRAK1-MECP2 region confer susceptibility to autoimmune thyroid diseases. *Molecular and Cellular Endocrinology*.

[17] Yan N., Meng S., Song R. H. (2015). Polymorphism of IL37 gene as a protective factor for autoimmune thyroid disease. *Journal of Molecular Endocrinology*.

[18] Fu Q., Zhao J., Qian X. (2011). Association of a functional IRF7 variant with systemic lupus erythematosus. *Arthritis and Rheumatism*.

[19] Paun A., Pitha P. M. (2007). The IRF family, revisited. *Biochimie*.

[20] Honda K., Taniguchi T. (2006). IRFs: master regulators of signalling by toll-like receptors and cytosolic pattern-recognition receptors. *Nature Reviews Immunology*.

[21] Mandac J. C., Chaudhry S., Sherman K. E., Tomer Y. (2006). The clinical and physiological spectrum of interferon-alpha induced thyroiditis: toward a new classification. *Hepatology*.

[22] Russo M. W., Fried M. W. (2003). Side effects of therapy for chronic hepatitis C. *Gastroenterology*.

[23] Burman P., Tötterman T. H., Öberg K., Anders Karlsson F. (1986). Thyroid autoimmunity in patients on long term therapy with leukocyte-derived interferon. *The Journal of Clinical Endocrinology & Metabolism*.

[24] Lin J. D., Wang Y. H., Liu C. H. (2015). Association of IRF8 gene polymorphisms with autoimmune thyroid disease. *European Journal of Clinical Investigation*.

[25] Goyal G., Panag K., Garg R. (2016). Prevalence of thyroid disorders in hepatitis C virus positive patients on interferon and antiviral therapy. *International Journal of Applied & Basic Medical Research*.

[26] Tomer Y. (2010). Hepatitis C and interferon induced thyroiditis. *Journal of Autoimmunity*.

[27] Tomer Y., Blackard J. T., Akeno N. (2007). Interferon alpha treatment and thyroid dysfunction. *Endocrinology and Metabolism Clinics of North America*.

[28] Bartalena L., Baldeschi L., Boboridis K. (2016). The 2016 European Thyroid Association/European Group on Graves’ Orbitopathy guidelines for the management of Graves’ orbitopathy. *European Thyroid Journal*.

[29] Khalilzadeh O., Noshad S., Rashidi A., Amirzargar A. (2011). Graves’ ophthalmopathy: a review of immunogenetics. *Current Genomics*.

[30] Han S., Zhang S., Zhang W. (2006). CTLA4 polymorphisms and ophthalmopathy in Graves’ disease patients: association study and meta-analysis. *Human Immunology*.

[31] Jurecka-Lubieniecka B., Ploski R., Kula D. (2014). Association between polymorphisms in the *TSHR* gene and Graves’ orbitopathy. *PLoS One*.

